# Targeting macrophages in liver fibrosis

**DOI:** 10.3389/fimmu.2026.1835489

**Published:** 2026-05-22

**Authors:** Lydia González del Barrio, David H. Ipsen, Dominik R. Pfister, Dora Hancz, Charlotte L. Scott

**Affiliations:** 1 Laboratory of Myeloid Cell Biology in Tissue Damage and Inflammation, VIB Center for Inflammation Research, VIB, Ghent, Belgium; 2 Department of Biomedical Molecular Biology, Faculty of Sciences, Ghent University, Ghent, Belgium; 3 Global Research, Novo Nordisk A/S, Bagsvaerd, Denmark

**Keywords:** heterogeneity, identity, kupffer cells, lipid-associated macrophages, liver fibrosis, macrophage-based therapies, macrophages, plasticity

## Abstract

Fibrosis results from excessive deposition of extracellular matrix (ECM) components following tissue injury. While initially protective, chronic injury drives pathological ECM accumulation, tissue remodeling, and organ dysfunction. In the liver, fibrosis represents a common endpoint of chronic diseases, including metabolic dysfunction–associated steatotic liver disease (MASLD). Macrophages are central players of the fibrotic hepatic niche, modulating inflammatory signals, immune regulation, and tissue repair. Recent advances demonstrate that hepatic macrophages form a heterogeneous and highly dynamic compartment, adopting diverse activation states that extend far beyond the classical M1/M2 paradigm. Importantly, liver fibrosis is now recognized as a potentially reversible process, with resolution being closely linked to the reprogramming of macrophages towards restorative phenotypes characterized by enhanced efferocytosis, reduced pro-inflammatory signaling, and increased matrix degradation capacity. As a result, efforts to design macrophage-based therapeutic strategies are shifting from non-specific approaches such as inhibition of monocyte recruitment towards approaches that actively promote pro-resolution programs, including targeted *in situ* reprogramming and more recently cell-based macrophage therapies. In this review, we summarize the hepatic macrophage landscape in the fibrotic liver and discuss current opportunities and challenges in developing macrophage-based anti-fibrotic therapies.

## Introduction

Fibrosis arises from a dysregulation of the wound-healing responses, leading to a remodeling of the ECM, loss of normal tissue architecture, and ultimately, organ dysfunction (1, 2). Although fibrosis is not classified as a disease, it represents a pathological hallmark common to a broad spectrum of chronic diseases across different organs, including the lungs (e.g., chronic obstructive pulmonary disease), kidneys (e.g., chronic kidney disease), heart (e.g., cardiac fibrosis), and liver (e.g., chronic liver disease) (3). Moreover, fibrosis is also a key factor for tumor development and metastasis, as neoplasms are also categorised as fibrotic diseases (4). In total, around one-third of all deaths worldwide are estimated to be caused by fibrotic diseases (5), demonstrating the public health burden posed by fibrosis. Despite sustained research and considerable advances, effective anti-fibrotic therapies remain limited.

Importantly, fibrosis is not a simple consequence of tissue damage, but rather a highly dynamic and complex process. Several cell types contribute to liver fibrosis, including hepatic stellate cells (HSCs) and other fibroblasts, liver sinusoidal endothelial cells (LSECs), and hepatocytes; however, the immune system also plays key roles (2, 6). Among all immune system components, macrophages are reported to be central regulators of the fibrotic microenvironment (7). First described in 1884 by Élie Metchnikoff, macrophages are multifunctional innate immune cells belonging to the myeloid lineage (8, 9). Distributed through all tissues, they act as professional phagocytes that recognize, engulf, and clear pathogens, apoptotic cells, and cellular debris. Macrophages are also key mediators of inflammation, immune regulation, and tissue repair. In fibrosis, macrophages have a pivotal role as regulators of pathology, where, depending on their activation state, they have been proposed to drive either fibrogenic or fibrolytic processes (7, 10). Given their central role in both fibrogenesis and fibrosis resolution, macrophages are emerging as key therapeutic targets for reversing fibrosis and restoring homeostasis. Thus, in this review, we focus on the macrophage landscape in the fibrotic liver, and we provide an overview of the current approaches to target macrophages for the treatment of liver fibrosis. As much of our current understanding of macrophage biology in liver fibrosis comes from studies in animal, predominantly murine models, much of this review will focus on these studies. However, whenever human data is available to either support or contrast the findings in animal models, this will be specifically highlighted.

## Macrophage heterogeneity and plasticity

Macrophages, found throughout the body in health and disease, are highly heterogeneous. This is reflected in both the co-existence of distinct macrophage populations within tissues as well as a degree of functional plasticity that allows individual macrophages to change their transcriptional and functional programs to adapt to diverse tissue environments and pathological contexts (11, 12).

In the steady state, macrophages populate virtually every tissue of the body, where they not only act as professional phagocytes for the clearance of apoptotic cells, microbes, and debris, but also carry out highly specialized functions adapted to their local microenvironment (13, 14). For example, microglia, the tissue-resident macrophages from the central nervous system, promote neuronal survival and are involved in synapse maintenance (15). On the other hand, tissue-resident macrophages from the adipose tissue are involved in insulin sensitivity control (16). Tissue-resident macrophages first appear during embryogenesis, where they can be derived from either yolk-sac macrophages or fetal liver monocytes (8, 17). Once these progenitors have colonized the organs, they expand locally and in most homoestatic tissues maintain their numbers throughout life through self-renewal (18). This has been demonstrated in multiple organs, including brain microglia, alveolar macrophages in the lung, and Kupffer cells (KCs) in the liver. In contrast, in other tissues such as the gut, skin, and heart, macrophages cannot efficiently maintain their numbers, leading to their constant replenishment over time (at different rates) by bone-marrow-derived monocytes (19). For more details, we refer you to the extensive reviews on this topic (19, 20).

Many tissue resident macrophages share key identity markers, including F4/80, CD64, and MERTK (21, 22). However, every tissue environment shapes the transcriptional programme of macrophages, leading to a unique expression of certain markers in different tissues (23, 24). For example, CLEC4F in murine liver KCs or SIGLECF in murine alveolar macrophages (25, 26). Upon injury or inflammation, circulating monocytes derived from bone marrow hematopoiesis can infiltrate into the tissues, where they differentiate into monocyte-derived macrophages (MDMs) and support, or even replace, tissue resident macrophages depending on the level of injury and persistence of damage (27).

Similar to their origins and markers, macrophages are also heterogeneous in terms of their functions. Mirroring the T helper classification (Th1/Th2), macrophages have historically been classified into M1 (also known as classically activated or pro-inflammatory macrophages) and M2 (alternatively activated or repair macrophages). This classification was based on *in vitro* studies where murine MDMs were stimulated with IFN-gamma/LPS or IL-4/IL-13, inducing a Th1 or Th2-like response, respectively (28). M1 macrophages were associated with a pro-inflammatory and antimicrobial profile producing TNF-α, IL-1β, and IL-12. In contrast, M2 were linked to a wound healing and tissue remodeling phenotype expressing IL-10, arginase-1, CD206, and secreting TGF-β (29). In the context of hepatic macrophages, M1 were proposed as the main drivers of inflammation and stellate cell activation, whereas M2 were described as the promoters of inflammation resolution and tissue remodeling (27, 28).

**Figure 1:**
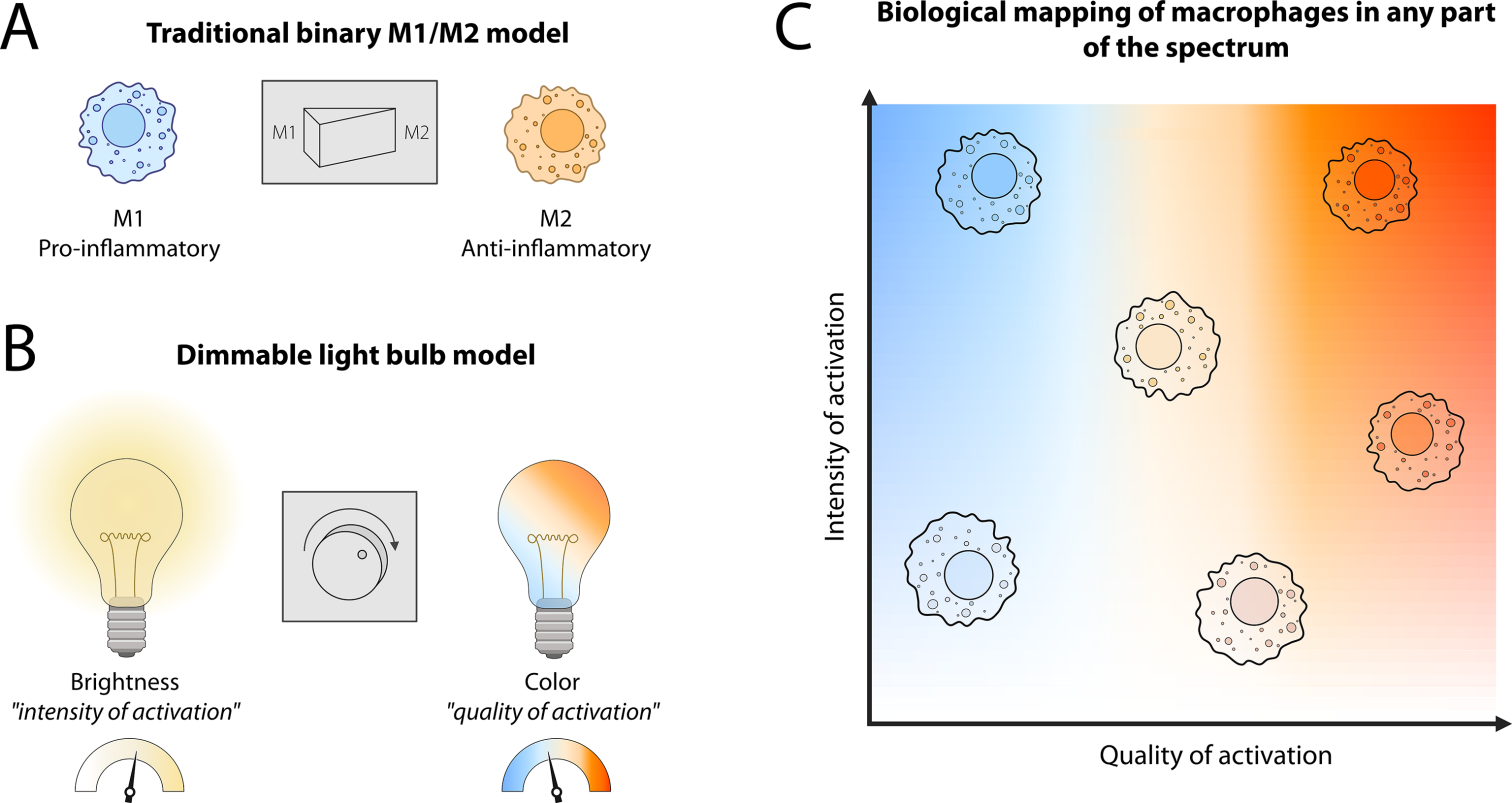
Conceptual analogy of macrophage activation as a light system. Macrophage activation in the fibrotic liver is a continuous, multidimensional spectrum rather than a binary M1/M2 switch, and this plasticity should be taken into account when designing macrophage-targeted therapies. **(A)** The traditional M1/M2 nomenclature represents macrophages as a binary switch, either “on” (M1, pro-inflammatory) or “off” (M2, anti-inflammatory). **(B)** Modern single-cell and functional studies show macrophage polarization is not binary, but rather a continuous spectrum of activation states better represented by a dimmable, color-changing light bulb: brightness reflects the intensity of activation, while color reflects the quality of responses. **(C)** Within fibrotic tissues such as the liver, macrophages can occupy different positions along this spectrum depending on microenvironmental signals.

While this binary classification has provided a useful framework to understand the opposing roles of macrophages in inflammation and tissue repair, we now know this classification dramatically oversimplifies macrophage activation *in vivo*, where macrophages are never exposed to a single signal, e.g., IFNγ, at any one time. The growing accessibility of single-cell transcriptomics has also revealed the presence of numerous subsets/states of macrophages in any given injured tissue in humans (30–33). Additionally, as tissue resident macrophages often die in response to severe injury and inflammation, being replaced by MDMs, it has been postulated that this perceived plasticity may merely be a consequence of replacing the resident macrophages with MDMs of an altered phenotype (21, 34). Addressing this question directly, more recent studies combining single-cell transcriptomics with fate-mapping studies, in both animals and humans, have revealed that some of the macrophage subsets identified during disease are activation states of tissue resident macrophages (35–38). This highlights that these cells do retain some plasticity and can adapt to the changing tissue microenvironment upon injury. Thus, these recent transcriptomic, proteomic, and epigenetic studies have demonstrated that macrophage activation should not be classified as an on/off light switch system (e.g., M1/M2), but rather as a continuous spectrum of activation states (10, 30). In this sense, macrophage activation can be better explained as a dimmable, color-changing light system. As a sliding dimmer, macrophages can modulate the “intensity” of their responses. At the same time, they can also adjust the “quality” of their activation. In the same way a light can shift from warm to cold tones, macrophages can combine different transcriptional and functional programs depending on the tissue microenvironment they are exposed to, becoming more pro- or anti-inflammatory as required (**Figure 1**).

With this recent evidence of plasticity, one of the major unresolved questions in macrophage biology concerns the fate of macrophages after activation. Several possibilities exist: (i) they may undergo apoptosis after fulfilling their function, (ii) they may persist in the tissue while maintaining their new phenotype, (iii) they may revert to their original functional state, (iv) they may adopt another phenotype (39). Moreover, it is likely that this fate will depend on the state of the tissue, e.g., differing if the injury is resolved compared with a state of chronic fibrosis. In murine models of fibrosis resolution, studies demonstrate that macrophage populations initially associated with fibrogenic functions can later contribute to ECM degradation and tissue repair (40, 41). Thus, these studies suggest functional reprogramming may occur at the population level. However, they do not fully resolve the extent to which macrophages reverse their phenotype or are replaced, nor do they detail which macrophages replace them. This distinction has important therapeutic implications. Macrophage depletion strategies may remove macrophage populations required for fibrosis resolution, while those approaches that promote macrophage reprogramming towards pro-resolution states could preserve beneficial functions and limit pathological inflammation. Thus, a detailed understanding of macrophage heterogeneity, plasticity, and fate during chronic liver disease is critical for designing macrophage-targeted anti-fibrotic therapies.

## Burden of liver fibrosis

Fibrosis is a key pathological feature of numerous, diverse chronic liver pathologies, including viral hepatitis (HBV and HCV), autoimmune liver disorders, cholangitis, alcohol-associated liver disease (ALD), and metabolic dysfunction-associated steatotic liver disease (MASLD), the hepatic manifestation of metabolic syndrome (42–44). MASLD represents a spectrum of disease states ranging from simple steatosis (a build-up of fat in the liver) to the more severe, inflammatory and fibrotic stages of disease termed metabolic dysfunction-associated steatohepatitis (MASH) (45). If unresolved, liver fibrosis can progress to cirrhosis, which is the term given to severe tissue scarring in the liver, which in turn is linked to higher chances of complications and mortality (42). The development of severe fibrosis and cirrhosis can also eventually lead to liver cancer (hepatocellular carcinoma; HCC). Together, liver pathologies contribute to a global mortality rate of more than 2 million deaths annually (46). Historically, viral hepatitis was the main cause of chronic liver disease. However, the prevalence of MASLD and MASH has drastically risen in the last few decades due to the increasing prevalence of obesity and type 2 diabetes. In contrast, thanks to the availability of vaccines, it is estimated that about 4% of the world’s population is currently living with viral hepatitis (hepatitis B and C viruses). Thus, MASLD is now the leading cause of chronic liver disease and hence liver fibrosis (47–49).

Determining the precise global prevalence of MASLD is challenging for several reasons. First, many patients with early-stage disease are undiagnosed due to their lack of symptoms. Furthermore, liver biopsy, the diagnostic gold standard, is invasive and declining in use, while the accuracy of non-invasive alternatives is highly variable. Lastly, differences in diagnostic criteria, age inclusion thresholds, and statistical methodologies further complicate the analysis. A systematic review and meta-analysis study from 1990 to 2019 estimated global MASLD prevalence in adults aged 20 and over at 30.05% (50). However, a more recent analysis from 2023, which reported age-standardised prevalence rates and hence accounted for differences in population age across countries, estimated an age-adjusted prevalence of 14.4% and a global prevalence of approximately 16.1% of the total population (51). Moreover, the predictions expect the prevalence to rise to a total of 1.8 billion cases by 2050 (51).

Despite this high burden of liver fibrosis worldwide, treatment options for patients remain limited. However, there is hope with two novel therapies being approved for patients since 2024. The first FDA approved therapy in 2024 was resmetirom. Resmetirom is an agonist of the thyroid hormone receptor-β (THR-β), which is indicated for MASH patients with moderate to advanced liver fibrosis, but not liver cirrhosis (52). The second drug to be approved in this realm was the GLP-1 analog, semaglutide, which was approved in 2025. As for resmitirom, semaglutide is indicated for MASH patients with moderate to advanced liver fibrosis, but not liver cirrhosis (53). While significant milestones in the treatment of MASH, these drugs need to be combined with diet and exercise to be effective and only address the needs of a subset of patients (54). Thus, more research and additional therapeutic strategies are needed to tackle the immense health burden that hepatic fibrotic diseases represent worldwide.

## Liver fibrosis

### The cellular architecture of the liver

Understanding the architecture and cellular dynamics of the liver is essential to untangling the mechanisms of liver fibrosis. The liver, the largest solid internal organ, is a highly vascularized and multifunctional organ responsible for several key processes in the human body, including detoxification, metabolism, immune regulation, and biosynthesis (55, 56). Encapsulated by the Glisson’s capsule, the liver parenchyma is mainly composed of hepatocytes organized into repetitive hexagonal units termed lobules. Each lobule is structured around a central vein, with several portal triads located at the periphery. A portal triad consists of three main structures: a branch of the hepatic artery supplying oxygenated blood from the systemic circulation, a branch of the portal vein bringing nutrient-rich blood from the gastrointestinal tract, and a bile duct lined by cholangiocytes, which are involved in bile modification and secretion. The blood enters the liver via the portal vein and hepatic artery, and flows from the portal triads toward the central vein through the liver sinusoids (55, 56).

Liver sinusoids are lined by fenestrated LSECs, which facilitate bidirectional exchange of molecules such as nutrients, metabolites, and plasma proteins between circulating blood and hepatocytes (57). The space of Disse, located between hepatocytes and LSECs and consisting mainly of collagen IV and elastin, is where the nutrient exchange occurs. It contains a population of fibroblasts, called HSCs, which upon their activation and transdifferentiation into myofibroblasts are key producers of ECM components (58, 59). KCs, the liver resident macrophages, are strategically located with at least part of their body within the liver sinusoid, acting as sentinels ready to respond to potential harmful elements present in the gastrointestinal tract-derived blood supplied by the portal vein (60, 61). Moreover, KCs extend multiple processes out of the sinusoids, with which they closely interact with HSCs (60, 61). These interactions are critical for KC identity and are also proposed to be required for maintaining HSCs in their quiescent state (62).

In addition to these complex cellular interactions, the liver exhibits a metabolic zonation due to gradients in oxygen and nutrients along the portal-central axis of each lobule. This results in the parenchymal cells of the liver, called hepatocytes, having different functions depending on their location along the sinusoid (63, 64). Each lobule consists of three distinct zones; zone 1 or periportal, closer to the portal triads, is rich in oxygen and the hepatocytes located here are involved in oxidative metabolism; zone 2 or mid-zonal, between the portal trials and the central vein with a mixed metabolic activity; and zone 3 or pericentral, closer to the central vein, is more hypoxic with hepatocytes here being more specialized in glycolysis, lipogenesis, and P450-mediated detoxification (63, 65). This zonation can also result in differences in the location of specific tissue injuries, which can also alter the generation and resolution of fibrosis. For example, in patients with metabolic dysfunction associated steatohepatitis (MASH), peri-central or zone 3 hepatocytes are more susceptible to injury, as these cells accumulate more lipids due to their lipogenesis specialization and are particularly sensitive to oxidative damage, both characteristics of MASH (66). Conversely, viral hepatitis predominantly affects peri-portal or zone 1 hepatocytes, as these are the first hepatocytes that the virus encounters when entering the liver (66).

Together, the considerable coordination between the different cells of the liver is key for the maintenance of tissue homeostasis. However, in response to chronic injury, this balance gets disrupted, leading to a pathological remodeling of the tissue architecture and cellular composition, resulting in immune dysregulation, HSC activation, and ultimately liver fibrosis (67).

### Pathology of liver fibrosis

**Figure 2:**
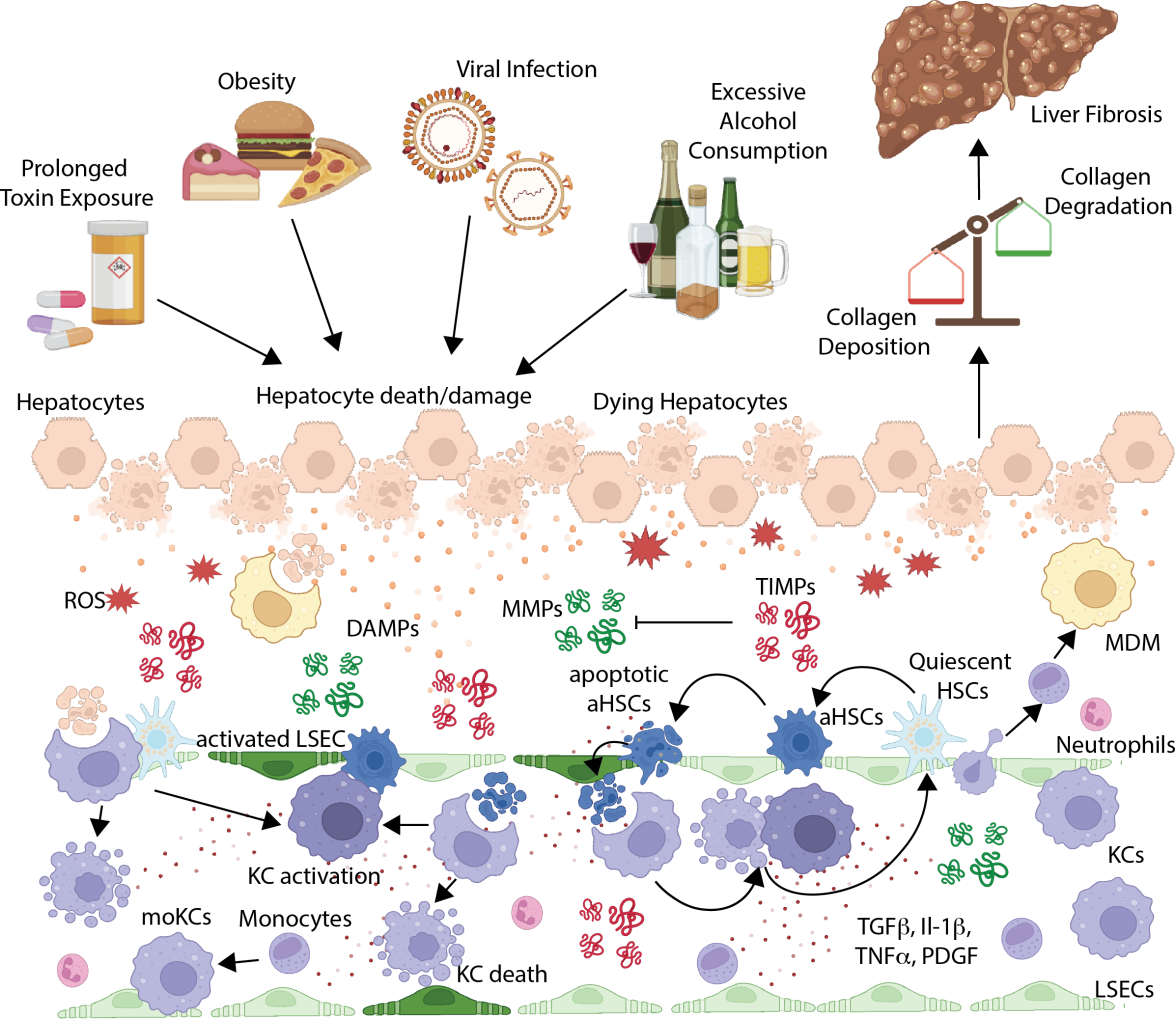
Cellular and molecular pathways in liver fibrosis. Liver fibrosis results from a complex multicellular cascade in which macrophages are proposed to be a central orchestrator, playing a dual role at both promoting and resolving fibrosis, depending on the context. Chronic liver injury caused by metabolic dysfunction–associated steatohepatitis (MASH)/obesity, viral hepatitis, prolonged toxin exposure, or excessive alcohol consumption results in hepatocyte damage and death. Injured hepatocytes release damage-associated molecular patterns (DAMPs) and reactive oxygen species (ROS), which activate resident Kupffer cells (KCs) and promote the recruitment of circulating monocytes that differentiate into monocyte-derived macrophages (MDMs) and monocyte-derived KCs (MoKCs), expanding the hepatic macrophage pool. In parallel, dead hepatocytes are phagocytosed by macrophages, which leads to either their activation and/or their death, depending on the context. Activated macrophages secrete pro-inflammatory and pro-fibrogenic mediators, including TNF-α, IL-1β, TGF-β, and PDGF, which drive the activation of quiescent hepatic stellate cells (HSCs) into collagen-producing activated HSCs (aHSCs). aHSCs are the principal source of extracellular matrix (ECM) proteins, leading to collagen deposition. Furthermore, aHSCs may undergo apoptosis, which will be cleared by KCs, again leading to KC activation and/or death, which further drives the activation of HSCs, resulting in a perpetuating cycle. Crosstalk between macrophages, aHSCs, (activated) liver sinusoidal endothelial cells (LSECs), and other recruited immune cells, such as neutrophils, sustain immune activation and fibrogenesis, while macrophage-induced aHSC apoptosis and phagocytic clearance can contribute to fibrosis resolution. Ultimately, fibrosis severity is determined by the balance between extracellular matrix (ECM) deposition by aHSCs and ECM degradation mediated by matrix metalloproteinases (MMPs), which are neutralized by tissue inhibitors of metalloproteinases (TIMPs).

Upon chronic liver injury, parenchymal and non-parenchymal cells undergo apoptosis, necrosis, or pyroptosis, releasing damage-associated molecular patterns (DAMPs) and other inflammatory mediators such as reactive oxygen species (ROS) (44, 68). DAMPs are recognised by pattern recognition receptors (PRRs), including toll-like receptors (TLRs), which are highly expressed on tissue resident immune cells, such as KCs in mice and humans (69). Recognition of DAMPs by KCs can lead to their activation, triggering a pro-inflammatory cascade by which they release pro-inflammatory cytokines and growth factors such as tumor necrosis factor alpha (TNF-α), and interleukin 1 beta (IL-1β), as well as chemokines like CCL2 (44, 70). These chemokines are released into the bloodstream and recognised by circulating monocytes and other immune cells, which will migrate to the liver and infiltrate into the area of injury. Once located in the murine or human liver, monocytes can differentiate into MDMs, including monocyte-derived KCs (MoKCs) and, if these MDMs get further activated, this can perpetuate liver inflammation amplifying the inflammatory cascade (44, 60, 71) (**Figure 2**).

In parallel, cytokines, including transforming growth factor beta (TGFβ), released by activated KCs and MDMs, together with ROS released by injured hepatocytes, activate the HSCs in the space of Disse (58). HSC activation represents a central event in liver fibrogenesis across species, as these cells are the main source of ECM proteins (**Figure 2**). Under homeostatic conditions, HSCs remain quiescent and store vitamin A in cytoplasmic lipid droplets. Upon activation, they transdifferentiate into a myofibroblast-like cell type. Activated HSCs (aHSCs) are characterised by an increased proliferative capacity, loss of lipid droplets, secretion of excessive ECM proteins, and release of ROS and pro-inflammatory cytokines and chemokines, promoting immune cell activation and creating a feedback loop that sustains fibrogenesis (72–75). Importantly, fibrosis is not only driven by an increased deposition of ECM components but also by the reduction of their degradation. Under homeostatic conditions, the liver maintains basal levels of ECM through a tightly regulated balance between the activity of matrix metalloproteinases (MMPs) and their inhibitors, known as tissue inhibitors of metalloproteinases (TIMPs) (76–78) and reviewed in (79). Both TIMPs and MMPs can be secreted by macrophages and aHSCs (80, 81). MMPs are a family of zinc- and calcium-dependent proteases that degrade several ECM components (**Figure 2**). Among the different classes of MMPs, the most relevant in the context of hepatic fibrosis are collagenases and gelatinases (80). Collagenases (e.g., MMP-1, MMP-8, MMP-13) degrade fibrillar collagens, mainly types I, II, and III, which are the major components of the fibrotic ECM. Gelatinases (e.g., MMP-12, MMP-9) degrade gelatines, laminin, and elastin (82). On the other hand, TIMPs are a family of endogenous proteins that inhibit the proteolytic activity of MMPs (**Figure 2**). Although four TIMPs are known (TIMP-1, -2, -3, and -4), TIMP-1 is the most relevant one in the context of liver fibrosis (81). TIMP-1 not only inhibits collagen degradation by suppressing MMP activity but also promotes survival and proliferation of aHSCs, perpetuating and amplifying the fibrotic response (80, 83).

One of the most promising developments in the field is the growing consensus that liver fibrosis can be a reversible process under certain conditions, particularly if the underlying cause of injury is removed (84–86). Resolution of fibrosis involves not only the degradation of ECM but also reprogramming of the immune microenvironment (87, 88). Macrophages are key in this process. Although macrophages are broadly activated during injury, liver macrophages comprise a heterogeneous and dynamic compartment (25, 31, 37). While the precise roles of the different subsets generated during injury and resolution are still under investigation, the prevailing dogma is that during active fibrogenesis, the macrophage pool is dominated by pro-inflammatory and pro-fibrogenic macrophages (31, 89, 90). It is tempting to speculate that these macrophages are likely primarily MDMs, but recent studies demonstrating that KCs can be activated under different settings mean that KC involvement cannot yet be ruled out (36, 91–94). Upon cessation of the injury, the hypothesis is that the macrophage compartment is reprogrammed towards a restorative phenotype, involving both the loss of the pro-fibrogenic macrophages and the (de)differentiation of macrophages into distinct restorative states. Current evidence (discussed below) suggests that these restorative macrophages mainly originate from MDMs (40, 90), although phenotype switching of pre-existing liver macrophages including MDMs recruited during active disease, cannot be ruled out. In any case, pro-resolution macrophages are functionally characterised by enhanced clearance of apoptotic cells, increased secretion of fibrolytic enzymes such as MMPs and cathepsins, and reduced pro-inflammatory signalling, thus promoting fibrosis resolution (95, 96). Understanding the landscape of liver macrophages and their functional characterisation is therefore crucial for the development of effective macrophage-targeted antifibrotic therapies.

## Hepatic macrophages and liver fibrosis

In the murine and human liver, macrophages are the most abundant immune cell type, representing around the 70% of all immune cells. This makes the liver one of the organs with the highest proportion of macrophages across the body (97). KCs occupy a unique strategic position within the sinusoidal lumen. In mice, KCs are predominantly found peri-portally, where they are constantly exposed to microbial products and food metabolites arriving from the gut via the portal vein (98). In this niche, KCs function as immune sentinels, maintaining the balance between tolerating harmless antigens that should not provoke an immune reaction and inducing a rapid defense response against pathogenic antigens (60, 61). While KCs are also found peri-portally in humans, recent evidence suggests they are more enriched in peri-central regions (99). Although the functional relevance of this remains to be determined, it has been postulated that this is to aid in the clearance of dying hepatocytes, which may turn over more rapidly in peri-central regions in humans due to higher metabolic activity (99). Across species, KCs are also proposed to play key roles in liver homeostasis, including clearance of senescent cells, cellular debris, and old erythrocytes, as well as endotoxins, immune complexes, and other potentially harmful molecules from the bloodstream (98, 100). In addition, KCs help to regulate iron and lipid metabolism and contribute to immune tolerance in the liver by producing anti-inflammatory mediators (60, 71, 98, 101). In addition to KCs, the liver contains a dynamic pool of macrophages derived from circulating monocytes. During homeostasis, monocyte contribution to KCs is limited, even in humans (102), and is rather restricted to the minor populations of capsule, central vein, and bile-duct macrophages (for more information on these minor populations, we refer to the review of liver macrophages in health and disease (60)). However, during chronic liver injury, large numbers of bone marrow monocytes are recruited to the liver, where they differentiate into MDMs, which can be found throughout the liver, including within the KC niche of the liver sinusoids. As such, MDMs represent a heterogeneous population that can adopt diverse functional states and fates depending on the inflammatory context, niche, and duration of injury (36, 37, 92, 94, 103–106).

Macrophage identity is often defined by the expression of specific surface and intracellular markers. Within the human liver, resident KCs are identified as *CD68^+^* macrophages co-expressing *VSIG4, TIMD4, CD163*, and *CD5L*, while non-KCs, including MDMs, capsule macrophages and bile-duct macrophages are identified as CD68^+^ cells lacking these KC markers (60, 62). Recruited MDMs initially express markers such as Ly6C (in mice), CCR2, and CX3CR1, but these markers are often transient, being lost as these cells differentiate from monocyte to macrophage (44, 60, 96, 105). MDMs that occupy the KC niche become what is termed monocyte-derived KCs (MoKCs), expressing all KC markers except TIM4, although as a maker of residency, this can also be acquired over time (25, 104). While specific markers have been considered to accompany activation status, whereby pro-inflammatory macrophages typically express high levels of CD86 and MHC-II, and reparative or anti-inflammatory macrophages often express CD163 and CD206 (107), marker expression regularly overlaps across activation states. As they can be dynamically regulated during injury and repair, the use of these markers to define macrophage functions should therefore be avoided. Indeed, in both the human and murine healthy liver, KCs (identified as VSIG4^+^TIM4^+^) express CD163, CD206, MHCII and CD86, demonstrating that these cells do not fit into either the pro-inflammatory or reparative categories based on these markers alone (62). Moreover, it has recently been shown that upon activation, resident murine KCs can lose expression of the canonical KC markers, including TIM4, VSIG4 and CLEC4F (91, 94). Thus, discrimination between hepatic macrophage subsets based on a restricted set of markers is unreliable, and ontogeny and niche context must be taken into account (96).

**Figure 3:**
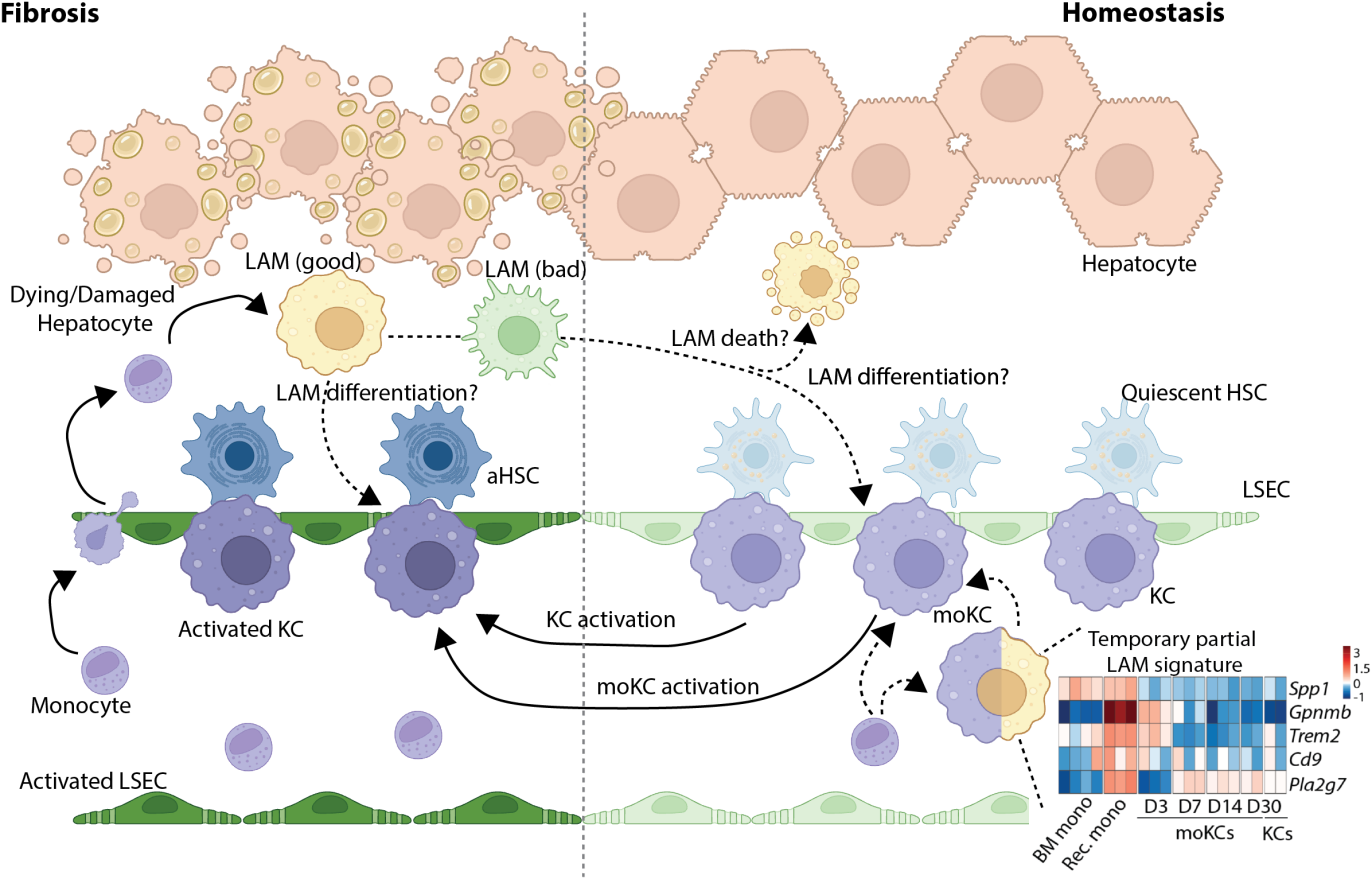
Remaining questions regarding LAM biology. Lipid-associated macrophage (LAM) biology remains incompletely understood. Fate-mapping tools and relevant human models are critically needed to resolve their pro vs anti-fibrotic roles, which will help to the development of future LAM-based therapeutic strategies. LAMs, also known as SAMs/NAMs, have been shown to be generated in the context of liver fibrosis, where they originate from recruited monocytes. These cells occupy a distinct niche from KCs, being located at zones of fibrosis. However, resident and monocyte-derived KCs can also acquire a LAM-like phenotype. Acquisition of the LAM/LAM-like phenotype has been shown to be, at least in part, dependent on the uptake and clearance of dying cells in the local microenvironment, suggesting that this may indeed reflect a conserved activation state of macrophages rather than a distinct subset. Interestingly, reanalysis of publicly available bulk RNA-seq data of monocytes entering the homeostatic liver and generating moKCs following depletion of their embryonic counterparts (25) has revealed that these cells can also temporarily acquire a LAM-like phenotype, whereby LAM-associated genes are upregulated in monocytes recruited to the liver (rec. monocytes, 1 day post KC depletion), compared with BM monocytes, and that this expression is maintained in the differentiating moKCs 3 days (D3) post KC deletion. By day 7, many of these genes have been downregulated to levels associated with embryonic KCs (KCs, D30), and this expression is maintained until at least 30 days post depletion (D30). The timing of this downregulation correlates with the acquisition of KC markers (D7) as previously described (25). This raises the question whether all BM monocytes transitioning into MDMs in the liver would go through a LAM intermediate stage or if this is associated only with a subset of MDMs? In line with this, the fate of LAMs during and following fibrosis resolution remains to be determined. Can LAMs exist in multiple flavours explaining data suggesting both pro and anti fibrotic roles for these cells? Moreover, while LAMs are no longer present in the liver upon return to homeostasis, it remains unclear if this is due to their death or differentiation into, for example, moKCs.

In the context of MASLD and MASH, a progressive loss of KCs, where the degree of loss is correlated with the severity of the injury, has been reported (36, 37, 104–106, 108). In mice, KC loss has been attributed to death via apoptosis (37, 106), although whether this holds true across all models of murine MASLD and in humans remains unclear. This loss of resident KCs is accompanied by the influx of monocytes to the liver, which can engraft and differentiate into MDMs. Those recruited to an intact empty KC niche become MoKCs, which do not express TIM4 initially, but can be acquired over time (104). On the other hand, those monocytes recruited to zones of steatosis and fibrosis appear to take on an alternative profile, which has been given multiple names, including lipid-associated macrophages (LAMs), scar-associated macrophages (SAMs) and NASH-associated macrophages (NAMs) (31, 104, 108, 109). Notably, these cells exhibit a very similar transcriptional profile, with the precise name (LAM, SAM, or NAM) reflecting the different studies in which these cells were identified. For the sake of clarity, here we will refer to all of these populations as LAMs. However, it is clear from this that macrophage nomenclature represents a major challenge in current macrophage research, and effort needs to be invested into the development of a consensus strategy for naming these cells (**Box 1**). LAMs in both mice and humans, are characterised by the expression of *TREM2, CD9, SPP1* (osteopontin), *FABP5*, and *GPNMB* (36, 109). Interestingly, this phenotype is not unique to the fibrotic liver, also being identified in other tissues in the context of sterile injury (32, 33, 38, 110–121) as well as in macrophages surrounding the bile duct in the healthy liver (62). Moreover, LAM-associated genes can also be detected in murine and human resident KCs upon sterile injury, e.g., MASH/fibrosis, and these activated KCs have been termed LAM-like KCs (36). Together, these findings argue against the LAMs being a distinct subset of macrophages, but rather support the idea that this may represent a functional state that can be adopted by any macrophage when exposed to specific microenvironmental signals, including lipids, apoptotic cells, and ECM components, irrespective of origin (**Figure 3**) (34, 97). In this regard, understanding the fate of LAMs in the fibrotic liver, becomes even more poignant. Does this state represent an end-point of differentiation, or do these cells retain plasticity and later convert their phenotype (**Figure 3**)? A re-examination of monocyte-KC development following resident KC depletion in mice has shown that monocytes entering the liver also temporally upregulate LAM-associated genes (although to a much lesser extent than observed in recruited MDMs in injury, **Figure 3**), raising the possibility that all macrophages may go through a LAM intermediate stage. Sophisticated fate mapping tools for the LAM-phenotype will be required to address these questions.

Box 1 **Towards a consensus strategy for macrophage nomenclature**

With the increasing affordability of single-cell transcriptomics, numerous studies have been conducted to investigate macrophage heterogeneity across a range of disease states. This has led to a better understanding of the different macrophage populations present, but has also led to many different names being given to macrophage subsets that appear across disease models, which causes confusion and prevents alignment between studies. A good example of this is the population of macrophages in the fibrotic liver, which have been called LAMs, SAMs, and NAMs in different studies, despite largely sharing a conserved transcriptional profile (31, 104, 108). Part of the reason for this is the pressure for novelty in publications, whereby claiming the presence of a new population of macrophages increases interest. The other main cause of this is the lack of a consensus regarding macrophage nomenclature. Importantly, this is not a question of semantics, as the names we give to cells can have significant consequences. For example, identifying a population of macrophages as pro-fibrotic in one tissue may make them an interesting target for therapeutic intervention. However, if the same population also fulfills a different function and is given a different name, then this overlap may be missed, and the therapy may fail, resulting in a waste of resources. So how should we name macrophage populations? Obviously here we need to come to a consensus as a field and no one group can decide this unanimously, however to start the discussion, we would propose that multiple aspects should be present in the name including the tissue where it is described, (putative) functions, ontogeny and the markers it expresses, which in our opinion, would allow cross context, tissue and species comparisons to be made between populations. Of course, all of this will lead to a long name, which makes it less attractive, thus we would propose to draw parallels with how humans are named in Western cultures. Firstly, we would propose a formal “legal” name for any macrophage, consisting of a title, first name, middle name, and surname. Here the title would detail the tissue in which the macrophage was identified, the first name would highlight 2–3 of the top marker genes for the population, the middle name would cover ontogeny, and the surname would detail (putative) functions and/or a historical name (e.g. for resident macrophage populations such as KCs, microglia, Langerhan’s cells etc). We suggest 2–3 top marker genes to enable a quick comparison between populations that does not rely on 1 marker, as the top gene may differ based on study design. Moreover, we highlight putative functions, as while ideally these would be confirmed functions, they are not always investigated in the initial studies describing the cells for one reason or another, including the lack of specific tools allowing the functions of the ‘new’ macrophages to be defined. To give concrete examples, this would lead to the names: Liver VSIG4^+^TIM4^+^ embryonically-derived Kupffer cells for what are now referred to as resident KCs, Liver TREM2^+^GPNMB^+^ monocyte-derived repair-associated macrophages for LAMs/SAMs/NAMs, Liver VSIG4^+^TIM4^-^ monocyte-derived Kupffer cells for moKCs, and Liver TREM2^+^GPNMB^+^ embryonically-derived (repair-associated) Kupffer cells for LAM-like KCs. However, as these names are long, we would then propose that after a first formal introduction, these names would be shortened to include only the first name and surname, leading to VSIG4^+^TIM4^+^ KCs, TREM2^+^GPNMB^+^ repair-associated macrophages (RAMs), VSIG4^+^TIM4^-^ KCs, and TREM2^+^GPNMB^+^ repair-associated KCs (RAKCs). Again, we want to emphasize that this is only a suggestion, designed to further stimulate discussion and an eventual consensus around this important topic.

The functional role of the distinct hepatic macrophage populations in the context of fibrosis is also an area of intense investigation, critical for the design of novel macrophage-based therapeutics and here also a number of questions still remain. For example, if the loss of resident KCs could be prevented, would this be beneficial? To date, considerable effort has been focused on the function of the LAMs, being the predominant macrophage population in the fibrotic liver. However, without tools to study these cells specifically, we still have more questions than answers. While the majority of murine studies to date have suggested a role for LAMs/LAM-like KCs in tissue repair, contributing to dead cell and lipid clearance, thus reducing lipotoxic stress, and preventing uncontrolled inflammation (36, 108, 122–126), these cells also produce mediators such as SPP1 and TGF-β that activate HSCs and hence may promote fibrogenesis (31). It remains unclear whether this activation is a beneficial fibrotic response required for tissue repair or whether it instead drives pathological fibrosis. The former option is supported by murine studies suggesting overexpression of *Spp1* in macrophages would ameliorate fibrosis and MASLD (127). However, evidence for the latter option also exists as, in mice, reduced fibrosis has been shown to correlate with the loss of LAMs (109). Thus, LAMs may have both protective and pathogenic roles, but exactly why this would be the case and how this would be regulated remains to be investigated. One hypothesis in this regard would be that the function of these macrophages may be dependent on the time spent in the fibrotic tissue. For example, the LAM phenotype may be induced to help clear debris and lipids, activating HSCs to induce tissue fibrosis needed for tissue repair, but with continued exposure to such signals, these cells may become overwhelmed and dysfunctional, leading to impaired clearance while continuing to produce pro-fibrotic mediators, exacerbating disease (**Figure 3**). Alternatively, different subsets of LAMs may exist with distinct functions. Thus, it is clear that additional studies are needed to address these outstanding questions. However, at this time, our ability to answer these questions is somewhat limited by the availability of tools and technologies. For example, to date, no LAM-specific Cre driver or inducible fate-mapping mouse models exist to selectively trace, deplete, or conduct gain/loss-of-function experiments with LAMs *in vivo* at defined disease stages. Such tools would allow us to test the temporal hypothesis, where LAMs could be initially protective but later become pathogenic, or the co-existence of distinct LAM subpopulations with opposing functions recruited at different times or from different precursors. Complementary approaches, such as CRISPR-based *in vivo* screens, could enable the modulation of LAM-associated genes and hence functions without the need for genetic models, however here the temporal element becomes more challenging. Moreover, strategies still need to be devised to specifically target guide mRNAs to LAMs, particularly in the absence of a LAM mouse model where *Cas9* expression could be limited to LAMs. This represents a key challenge given the highly phagocytic nature of the different hepatic macrophage populations. Indeed, previous studies in mice using *ex vivo* isolated macrophage populations have shown that KCs are considerably more phagocytic than LAMs (36). Beyond animal models, human liver organoid co-culture systems combined with CRISPR-based *in vitro* technologies could shed some light on the roles of LAMs, however here the bottleneck is the generation of *bona fide* LAMs *in vitro*. Current evidence suggests a role for efferocytosis in their generation (36), however, a more in-depth understanding of these cells and for example the transcription factors governing their identity will likely be required to engineer these cells effectively *in vitro*. Thus, taken together, these studies and our current technical limitations highlight the need for further research efforts into these populations before we can really start to think about deploying LAM-targeted therapies.

## Designing macrophage-based therapies for liver fibrosis

**Figure 4:**
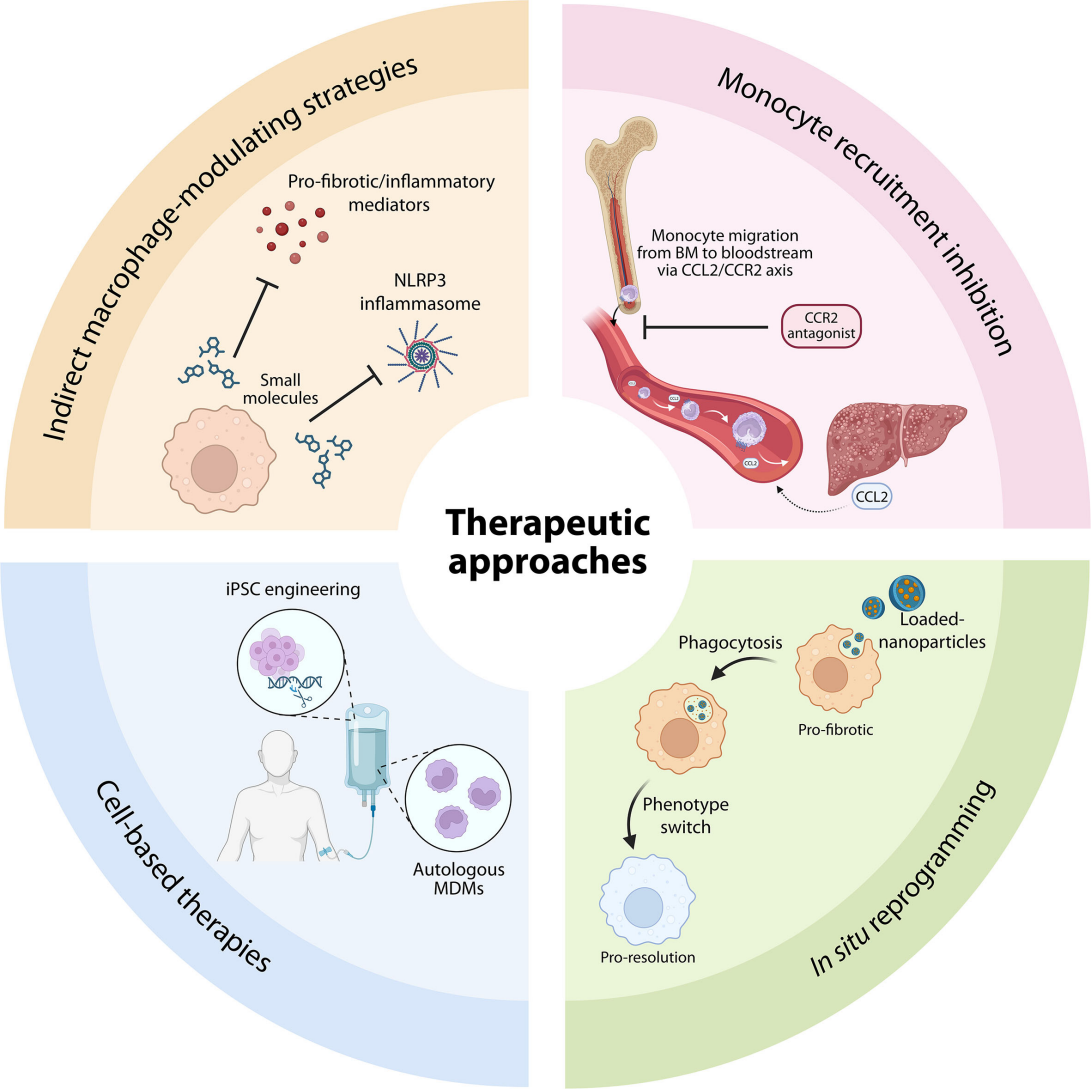
Anti-fibrotic macrophage-based therapeutic approaches. Overview of current and emerging strategies to target macrophages for the treatment of liver fibrosis. Therapeutic approaches are grouped into four main categories. Indirect macrophage-modulating strategies aim to inhibit pro-fibrotic and inflammatory mediators or signalling pathways, such as cytokine production or inflammasome activation. Monocyte recruitment inhibition limits the influx of circulating monocytes into the injured liver, for example, through blockade of the CCL2-CCR2 axis. *In situ* macrophage reprogramming seeks to shift endogenous macrophages towards a pro-restorative phenotype using targeted delivery systems, such as loaded nanoparticles. Cell-based therapies involve the administration of autologous monocyte-derived macrophages or engineered induced pluripotent stem cell (iPSC)-derived macrophages to promote tissue repair and fibrosis resolution. To date, no macrophage-targeted strategies have achieved robust clinical efficacy. Future progress will likely require combining the distinct strategies.

The growing understanding of macrophage heterogeneity, plasticity, and fates in liver fibrosis has opened multiple therapeutic opportunities aiming at modulating macrophage numbers, origins, or functions. Targeting macrophages in fibrosis is not a new concept. Early anti-fibrotic strategies in animal models, mainly murine, focused on neutralizing macrophage-derived mediators such as transforming growth factor-β (TGF-β), tumor necrosis factor-α (TNF-α), or inflammatory chemokines that drive immune cell recruitment (95, 128–132). However, the growing evidence that macrophages perform both pro-fibrotic and pro-resolution functions depending on context has reshaped how we think about macrophage-based therapeutics. As a result, the current central challenge in macrophage-based therapy design is to decipher which macrophage programs should be modulated, at what time, and through which mechanisms, to achieve fibrosis resolution without compromising tissue repair or host defense. Current approaches to developing macrophage-based therapeutic strategies for liver fibrosis can be divided into four broad categories: (i) indirect macrophage-modulating strategies, (ii) inhibition of macrophage recruitment, (iii) *in situ* macrophage reprogramming, and (iv) cell-based macrophage therapies (**Figure 4**, **Table 1**), and these are discussed below.

**Table 1:** Overview of macrophage-targeted therapeutic strategies for liver fibrosis.

Strategy	Target/Mechanism	Key Evidence	Pros	Limitations	Potential Improvements
Indirect macrophage modulation	Inhibition of pro-inflammatory pathways (TGF-β, NLRP3, TNF-α)	Preclinical + limited clinical	Established targets, pharmacologically accessible	Poor cell specificity, systemic toxicity, interferes with repair	Liver-targeted delivery, macrophage specific uptake
Recruitment blockage	CCL2–CCR2 axis	Strong preclinical, failed phase III	Reduces inflammatory monocyte influx	Blocks reparative macrophages, time sensitive	Temporal or partial blockade, time-specific use
*In situ* reprogramming	Nanoparticles, nuclear receptors, immunometabolism	Robust preclinical	Macrophage maintenance, aligns with plasticity	Delivery specificity, durability of reprogramming	Spatial targeting, combined metabolic and signaling approaches
Cell-based macrophage therapy (autologous)	*Ex vivo* polarized macrophages	MATCH phase I/II	High specificity, low rejection risk, can be genetically modified	Phenotype instability, poor and expensive scalability	Intrinsic stabilization, engineered macrophages
Stem cell-derived macrophages	Off-the-shelf macrophages	Preclinical	Scalable, standardized, can be genetically modified	Immunogenicity, phenotype instability, cost	Use of hypoimmunogenic stem cells

### Indirect macrophage-modulating strategies

This approach aims to target molecules produced by the macrophages rather than the macrophages themselves. However, while macrophages are an important source of pro-fibrotic mediators during liver injury, they are often not the only cells producing such mediators or the targets of these mediators, which means that most therapies targeting these pathways do not act specifically on macrophages and, as a result, can have adverse effects. For example, while macrophages contribute to TGF-β production during early inflammatory stages, across species activated HSCs become the dominant source of TGF-β as fibrosis progresses (133, 134). Consistent with this, the small molecule pirfenidone (PFD), has been shown to reduce TGF-β signaling and inflammatory cytokine production in different murine experimental systems (135–137), but its main anti-fibrotic efficacy in the liver is mostly attributed to suppression of HSC activation (138, 139). While clinical studies in patients with compensated cirrhosis demonstrated acceptable safety and modest anti-fibrotic effects (140), these studies also highlighted the limitations of systemic pathway inhibition as systemic TGF-β blockade was associated with immune dysregulation, impaired tissue homeostasis, and increased oncogenic risk, limiting broader clinical application (141). Similarly, while inhibition of inflammasome signaling, particularly NLRP3 activation in myeloid cells, demonstrated promising anti-fibrotic effects in murine models (142–144), translation to humans has faced significant challenges. GDC-2394, a systemic small-molecule NLRP3 inhibitor, demonstrated liver toxicity in first-in-human studies, leading to early trial termination (145, 146). Collectively, these studies indicate that a substantial limitation of indirect macrophage-modulating strategies lies in their low cellular and tissue specificity. As such, future improvements will depend on limiting pathway inhibition to the fibrotic liver, for example, by using liver-targeted modalities that specifically target liver macrophages. Without these improvements, systemic inhibition of shared pro-fibrotic pathways is likely to remain limited due to side effects.

### Inhibiting monocyte recruitment to reduce MDMs

One of the most explored strategies to limit fibrogenesis has been the inhibition of monocyte recruitment to the injured liver by targeting the CCL2–CCR2 axis. CCL2 is highly upregulated in chronic liver injury and acts as an important chemoattractant for CCR2^+^ inflammatory monocytes (147), the progenitors for MDMs. Early studies showed how genetic deletion of CCR2 in knockout murine models reduced hepatic monocyte infiltration, attenuating inflammation and limiting fibrosis progression (89). These preclinical findings inspired the clinical application of cenicriviroc (CVC) for MASH treatment in patients. CVC is a dual CCR2/CCR5 antagonist initially developed for HIV and tested successfully on murine models of liver fibrosis and MASH. These preclinical studies demonstrated a significant reduction in collagen deposition (148). In the same line, in phase IIb clinical trials, CVC achieved improvement in patients with advanced fibrosis (149). However, phase III studies did not confirm the previous results, as they failed to show significant improvement of fibrosis without worsening steatohepatitis in the first year of treatment in adults with MASH (150). Thus, these findings highlight an important limitation of the strategies aiming at blocking the recruitment of monocytes, whereby a broad and long-term blockade of monocyte recruitment may prevent endogenous repair mechanisms in the liver. This supports the idea that MDMs are not exclusively pathogenic but are also essential for tissue repair and fibrosis resolution, as discussed above. Thus, a deeper understanding of the recruitment dynamics and the functional profiles of distinct MDM subsets and states could therefore help in the development of more precise and context-dependent therapies. For example, blocking the recruitment during phases dominated by pro-fibrogenic macrophages while preserving the recruitment during resolution steps may achieve fibrosis regression without compromising tissue repair.

### *In situ* macrophage reprogramming

In contrast to strategies based on recruitment blockade, *in situ* macrophage reprogramming aims to directly modulate the functional state of endogenous macrophages within the injured liver. Rather than eliminating macrophages or preventing their accumulation, this strategy aims to shift macrophages from pro-fibrotic and inflammatory states towards restorative, pro-resolution phenotypes, while preserving their essential roles in host defense, efferocytosis, and tissue repair. This approach better reflects macrophage biology, aligning with their dynamic and reversible nature, and offers the possibility of promoting fibrosis resolution without impairing their longevity. One of the most explored pre-clinical approaches for *in situ* macrophage reprogramming relies on targeted delivery of immunomodulatory agents to liver macrophages, including small-molecule drugs and nucleic acids (151–154). This approach takes advantage of the strong phagocytic capacity of macrophages, as well as the accumulation of nanoparticles and liposomes in the injured liver. Experimental studies using murine models have shown that intravenously administered liposomes and polymeric nanoparticles selectively accumulate in hepatic macrophages compared with other liver cell types (155, 156). In several preclinical models of liver fibrosis, nanoparticle-based systems delivering small molecules, siRNAs of microRNAs have successfully modulated macrophage activation states and reduced fibrosis progression (151, 157–159). For example, dexamethasone-loaded nanoparticles have shown to reprogram macrophages towards a more anti-inflammatory and restorative phenotype *in vitro*, and reduced activation of HSCs and collagen deposition in a chronic liver injury mouse model (160). However, other studies in rat models failed to demonstrate comparable anti-fibrotic effects and instead reported increased collagen deposition despite effective suppression of inflammatory signaling (161), suggesting this approach may suffer from a strong context and model dependency.

More recently, macrophage immunometabolism has emerged as a powerful tool for *in situ* reprogramming. *In vitro* pro-fibrotic macrophages mainly depend on glycolysis, while reparative macrophages depend more on oxidative phosphorylation and fatty acid oxidation (162). Pharmacological modulation of these metabolic pathways can shift macrophage function towards pro-resolution states and is thus currently being explored in pre-clinical models. Peroxisome proliferator-activated receptors (PPARs) are nuclear transcription factors involved in lipid metabolism and energy homeostasis, and play an important role in controlling macrophage inflammatory responses. In experimental models of liver fibrosis, mainly murine CCl_4_ and diet-induced MASH models, activation of PPARγ reduced inflammatory cytokine production in macrophages, limited HSC activation, and attenuated fibrosis progression (163, 164). Some PPAR agonists have been tested in clinical trials, such as elafibranor and lanifibranor. Both have been tested successfully on phase II clinical trials, proving they are well-tolerated by patients and showing MASH resolution without worsening of fibrosis (165, 166). However, while phase III for lanifibranor is still ongoing, the same phase trial for elafibranor failed due to a lack of robust anti-fibrotic effects (NCT identifier 02704403). Moreover, the systemic administration of these compounds raises concerns about potential adverse effects, highlighting the difficulty of translating these approaches into the clinic.

In line with this idea, in the context of MASH and MASLD, where macrophages are chronically exposed to excess lipids, dying cells, and fibrotic ECM, improving macrophage lipid handling and mitochondrial health may reduce lipotoxic stress, limit inflammatory activation, and indirectly slow the fibrotic responses of aHSCs. To this end, TREM2 has emerged as an interesting target for manipulation. As explained above, TREM2 is induced in injury-adaptive macrophage states (LAMs) across multiple tissues, including liver, adipose tissue, and brain. Direct pharmacological modulation of TREM2 signaling *in vivo* remains in early stages, with Alzheimer’s disease as the setting in which it is most advanced (167–170). However, in experimental liver fibrosis in murine models, TREM2-deficient macrophages failed to acquire reparative phenotypes, showed impaired efferocytosis, and exacerbated fibrogenesis (36, 122–126). These findings support the idea that boosting endogenous reparative macrophage programs, as driven by TREM2 signaling, may promote fibrosis resolution more effectively than only preventing macrophage accumulation.

### Cell-based macrophage therapies

Due to the clinical success of chimeric antigen receptor (CAR) T cells in blood cancer treatments, the immune cell-based therapy field is growing rapidly. More recently, CAR macrophages have been proposed for the treatment of solid tumors (171). Beyond oncology, the central role of macrophages in regulating inflammation, tissue repair, and remodeling has generated strong interest in moving macrophage-based cell therapies to regenerative medicine. Cell-based macrophage therapies provide a direct approach to deliver pro-regenerative macrophages to the diseased liver. Preclinical studies have shown that infusion of *ex vivo* polarized macrophages, especially those shifted towards an ECM-degrading, anti-inflammatory phenotype, can reduce fibrosis and enhance tissue repair in murine models (172), providing initial evidence regarding the viability of this approach and opening the door for potential translation. Importantly, the mechanisms of action of such macrophage cell therapies remain unknown, with the possibility existing that the effects are due to their regulation of the endogenous macrophages rather than intrinsic regulation of the transferred cells (173, 174). Thus, we still have much to learn about such strategies. Nevertheless, current cell-based macrophage therapies can be broadly divided into two categories: (i) autologous macrophage-based therapies and (ii) stem cell-derived macrophage therapies. The former involves the isolation of monocytes from the patient’s own blood, followed by their ex-vivo differentiation into macrophages before reinfusion into the same patient, while in the latter, macrophages are generated from stem cells (175).

The first clinical evidence for macrophage-based therapy in liver disease comes from the MATCH (Macrophage Therapy for Cirrhosis) program, led by the University of Edinburgh. In this program, autologous MDM infusions were administered to patients with compensated cirrhosis. It is important to note that the preclinical studies preceding the MATCH trial used models of acute fibrosis (mainly CCl_4_ murine models), while typical human cirrhosis is established in a chronic manner. If macrophage infusion can achieve comparable anti-fibrotic effects in humans, is still therefore, an open question that the MATCH study has only started to resolve. Phase I studies demonstrated that macrophage infusion was safe and well-tolerated, and even showed improvement in some serum biomarkers after treatment (C3M, PRO-C3, and ELF) (176). Unfortunately, the phase II studies did not meet the primary endpoint, not being able to demonstrate an improvement in the Model for End-Stage Liver Disease (MELD) score in a period of 90 days after macrophage infusion. However, several limitations of the study were reported, such as the endpoint sensitivity, the study power, and even the trial design due to ethical limitations to maintain participant blinding. An important design limitation of the MATCH study concerns the use of the MELD score as the primary endpoint (177). Since the trial was designed and initiated, the field has recognised that the MELD score is not the most optimal endpoint for evaluating therapeutic efficacy in trials targeting fibrosis progression and resolution (177, 178), and what are now considered the best endpoints, including (transplant-free) survival were treated as secondary outcomes in the MATCH phase II study (179). Thus, the absence of statistically significant effects may reflect the limitations of the study design rather than a lack of biological activity, as longer-term results showed that treated patients developed fewer liver-related adverse effects and improved survival compared to the control group, suggesting potential clinical benefits (180, 181). These results provided an initial proof of concept that macrophage infusion can activate reparative pathways in advanced liver disease, and led to the development of RTX001, an engineered regenerative autologous macrophage therapy. Autologous monocyte-derived macrophages are genetically modified *ex vivo* to enhance the expression of IL-10 and matrix metalloproteinase-9 (MMP-9), thereby combining anti-inflammatory and matrix-degrading functions (182). RTX001 represents a next-generation macrophage therapy designed to improve both potency and phenotype stability. Pre-clinical data presented at international meetings suggest that RTX001 has higher anti-inflammatory and anti-fibrotic activity compared with non-engineered macrophages in mice, supporting their potential to promote fibrosis resolution. Early clinical evaluation of RTX001 is currently ongoing in patients with decompensated cirrhosis (EMERALD study), representing the first engineered regenerative macrophage therapy to be tested in humans (183).

Despite the promising outcomes, autologous cell therapies represent a manufacturing challenge. While the use of patient-derived cells minimizes the risk of immune rejection, this also limits scalability and increases production costs, as each therapy must be produced individually for a single patient (175). Induced pluripotent stem cell (iPSC)-derived macrophages have therefore emerged as a scalable and programmable alternative to autologous cell therapies. iPSC-derived macrophages can be manufactured in large quantities, cryopreserved, and distributed as off-the-shelf products. Importantly, these cells closely match tissue macrophages at both transcriptional and functional levels (184–186). Recent advances in bioreactor-based production platforms allow standardized, GMP-compatible manufacturing with considerable yield and phenotype consistency, allowing the production of multiple doses from a single production batch (187). iPSC-derived macrophages can also be genetically engineered to boost pro-resolution functions and/or reduce pro-fibrotic pathways. Despite these advantages, several challenges remain. Allogeneic administration raises concerns related to immune rejection, which may require host conditioning or immunosuppression. An alternative option would be the use of hypoimmunogenic stem cells, which are modified stem cells designed to evade the immune system of the host by eliminating the expression of highly immunogenic molecules, such as HLA class I and/or II. This approach would reduce the risk of immune rejection during the infusion, while enabling the generation of “off-the-shelf” therapies that could be used across multiple patients (188).

While macrophage-based cell therapies are gaining traction, key concerns remain. Firstly, what does a *bona fide* anti-fibrotic macrophage look like? Moreover, if engineered, will this macrophage phenotype remain stable once administered to patients, given the sensitivity of macrophages to environmental signals? Although the plasticity of macrophages is essential for their normal function, it makes their therapeutic use very complex. When macrophages are introduced in inflamed tissues, such as the fibrotic liver, they may rapidly shift their reparative phenotype towards a pro-inflammatory phenotype, reducing their efferocytosis ability, ECM remodeling, and exacerbating inflammation. For this reason, specific mechanisms to stabilize their original functional state will need to be introduced to avoid undesirable phenotype changes (189, 190). Alternatively, if the response is not mediated by the transferred macrophages but rather their effects on the endogenous population, such stabilisation strategies may not be required. Thus, together it is clear additional basic research is required to understand macrophage phenotypes and plasticity as well as the precise modes of action involved to be able to optimise this approach. Lastly, additional open questions include engraftment efficiency, long-term persistence in the tissue of interest, and safety (191). This, coupled with the high costs involved in the production of such cell-based therapies (192), is currently limiting their use in the clinic.

## Current limitations and future perspectives

Despite rapid progress and many advances in our understanding of macrophage biology in liver fibrosis, several challenges keep limiting the translation of macrophage-based therapies into effective clinical treatments. Firstly, the field’s reliance on animal, particularly mouse, models likely represents a big hurdle in the path towards effective therapies. Indeed, there have already been many discrepancies between preclinical and clinical outcomes across the different therapeutic approaches discussed above, where robust anti-fibrotic effects observed in murine models (e.g. CCl_4_, bile duct ligation, or diet-induced MASH models) have frequently failed to translate into significant clinical benefit (193–199). This likely reflects the differences in disease mechanisms, cell composition, and mechanistic pathways between rodents and humans, highlighting the need for better human-relevant validation systems. Outside of species differences, another key limitation is our still incomplete understanding of macrophage fate and functional states of dynamic macrophage populations in chronic injury. While single-cell and spatial transcriptomic studies in mice have revealed a remarkable diversity of macrophage activation states, it remains unclear if these states represent stable phenotypes or transient responses to microenvironmental signals. Moreover, comparable datasets with samples from patients of liver fibrosis resolution are currently not available to translate these findings into humans. Resolving if pro-fibrotic and pro-resolution macrophages develop from different lineages or from reversible state transitions within the same populations will therefore be key for future therapeutic design. Similarly, understanding how this is regulated over the course of disease progression and resolution, will also be critical, allowing strategies to be developed which alter pro-fibrotic macrophages, but not at the expense of tissue repair. Importantly, recent pre-clinical evidence suggests that macrophage programs are important for disease outcome. The adoption of a shared activation state, such as the LAM program, by KCs and MDMs, and the functional redundancy between these populations, highlights the relevance of such programs. Thus, we believe that future therapies should focus less on selectively depleting or replacing specific macrophage subsets and more on boosting beneficial restorative programs, such as efferocytosis, ECM degradation, and inflammation resolution, either in transferred or endogenous macrophages. Bringing together single-cell biology, fate mapping approaches, spatial omics, and functional experiments will be key to identifying macrophage states that can be targeted for therapy. Using this knowledge together with precise drug-delivery tools and a better knowledge of time-context intervention will likely allow us to move towards the generation of new, more precise, safe, and efficient macrophage-based therapies.
